# Some Basic Aspects of HLA-G Biology

**DOI:** 10.1155/2014/657625

**Published:** 2014-04-09

**Authors:** Estibaliz Alegre, Roberta Rizzo, Daria Bortolotti, Sara Fernandez-Landázuri, Enrico Fainardi, Alvaro González

**Affiliations:** ^1^Department of Biochemistry, University Clinic of Navarra, Pamplona, Spain; ^2^Department of Medical Sciences, Section of Microbiology and Medical Genetics, University of Ferrara, Ferrara, Italy; ^3^Neuroradiology Unit, Sant'Anna Hospital, Ferrara, Italy

## Abstract

Human leukocyte antigen-G (HLA-G) is a low polymorphic nonclassical HLA-I molecule restrictively expressed and with suppressive functions. HLA-G gene products are quite complex, with seven HLA-G isoforms, four membrane bound, and other three soluble isoforms that can suffer different posttranslational modifications or even complex formations. In addition, HLA-G has been described included in exosomes. In this review we will focus on HLA-G biochemistry with special emphasis to the mechanisms that regulate its expression and how the protein modifications affect the quantification in biological fluids.

## 1. Introduction

Human leukocyte antigen-G (HLA-G) is a major histocompatibility complex class I antigen encoded by a gene on chromosome 6p21. It differs from classical HLA class I molecules by its restricted tissue distribution and limited polymorphism in the coding region. HLA-G role in immune tolerance was uncovered studying its expression in trophoblast cells at fetus-maternal interface [1]. Several studies have found an aberrant or reduced expression of both HLA-G mRNA and protein in pathological conditions such as preeclampsia [2] or recurrent spontaneous abortion [3] in comparison with normal placentas. HLA-G expression has been documented in few tissues during physiological conditions, such as cornea, thymus, erythroid, and endothelial precursors [4–6], and in a variable percentage of serum/plasma samples from healthy subjects [7] where the main producers seem to be activated CD14+ monocytes [8]. An ectopic expression of HLA-G molecules has been observed during “no-physiological” conditions, such as viral infection [9–12], cancer [13], transplantation [14–18], and in inflammatory and autoimmune diseases [19–21]. Thus, a growing body of evidence has indicated HLA-G as a suitable key actor in different pathologies. In fact, HLA-G may exhibit two distinct effects in pathological conditions: it could be protective in inflammatory and autoimmune diseases [22] or it could be dangerous, for example, in tumors or infectious diseases.

## 2. HLA-G Expression and Regulation

**Figure 1:**
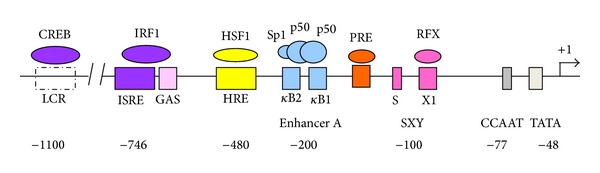
HLA-G unique promoter region. Enhancer A element (*Κ*B1, *Κ*B2, Sp1): NF-*Κ*B; interferon-stimulated regulatory element (ISRE); interferon regulatory factor (IRF); interferon-gamma activated site (GAS); SXY region; progesterone response element (PRE); hypoxia response element (HRE).

The HLA-G production is controlled by several polymorphisms both in the promoter and in the 3′ untranslated region (3′ UTR) that modify the affinity of gene targeted sequences for transcriptional or posttranscriptional factors, respectively [24]. Twenty-nine single nucleotide polymorphisms (SNPs) have been identified in the HLA-G promoter region, which may be involved in the regulation of HLA-G expression, considering that many of these polymorphisms are within or close to known or putative regulatory elements (Figure 1). The* HLA-G* 5′ upstream regulatory region (URR) is unique among the* HLA* genes [25] and is unresponsive to NF-*κ*B [25] and IFN-*γ* [26], due to the presence of a modified enhancer A (enhA) and a deleted interferon-stimulated response element (ISRE). A locus control region (LCR) located −1.2 kb from exon 1 exhibits a binding site for CREB1 factor, which also binds to two additional cAMP response elements at −934 and −770 positions from the ATG start codon. In addition, an ISRE for IFN response factor-1 (IRF-1) is located at the −744 bp position [24] and is involved in* HLA-G* transactivation following IFN-*β* treatment [27]. The* HLA-G* promoter also contains a heat shock element at the −459/−454 position that binds heat shock factor-1 (HSF-1) [28] and a progesterone receptor binding site at −37 bp from ATG start codon [29]. Several promoter region polymorphisms coincide with or are close to known or putative regulatory elements and thus may affect the binding of* HLA-*G regulatory factors [30]. The −725 C>G/T SNP is very close to ISRE, and the −725 G allele is associated with a significantly higher expression level compared with the other alleles [31]. The polymorphic sites at the 5′ URR are frequently in linkage disequilibrium (LD) with the polymorphic sites identified at the 3′ UTR, some of them influencing alternative splicing and mRNA stability [25].

A 14 base pair (14 bp) insertion/deletion (INS/DEL) polymorphism (rs66554220) in exon 8 involves mRNA stability and expression [32]. In particular, the DEL allele stabilizes the mRNA with a consequent higher HLA-G expression [33, 34]. The presence of an adenine at position +3187, modifying an AU-rich motif in the* HLA-G* mRNA, decreases its stability [35]. One single nucleotide polymorphism (SNP) C>G at the +3142 bp position (rs1063320) has been explored by Tan and coauthors [36]. The presence of a guanine at the +3142 position may influence the expression of the HLA-G locus by increasing the affinity of this region for the microRNAs miR-148a, miR-148b, and miR-152, therefore decreasing the mRNA availability for translation by mRNA degradation and translation suppression. The influence of the +3142G allele has been demonstrated by a functional study in which HLA-G high-expressing JEG-3 choriocarcinoma-derived cells have been transfected with miR-148a, decreasing soluble HLA-G levels. The contrasting results obtained by Manaster and coauthors [37], who have reported the absence of +3142 C>G effect on the miRNA control of membrane HLA-G expression, prompt further considerations on the relationship between this polymorphism and membrane HLA-G expression. Other SNPs have been identified as implicated in miRNA interaction. In particular, +3003, +3010, +3027, and +3035 SNPs are influenced by miR-513a-5p, miR-518c*, miR-1262 and miR-92a-1*, miR-92a-2*, miR-661, miR-1224-5p, and miR-433 miRNAs [35]. The miR-2110, miR-93, miR-508-5p, miR-331-5p, miR-616, miR-513b, and miR-589* miRNAs target the 14 bp INS/DEL fragment region, and miR-148a, miR-19a*, miR-152, mir-148b, and miR-218-2 also influence the +3142 C/G polymorphism.

HLA-G is a stress-inducible gene: heat shock, hypoxia, and arsenite increase different HLA-G alternative transcripts [28, 38]. The indolamine 2,3-dioxygenase (IDO), an enzyme which metabolizes tryptophan, induces HLA-G expression during monocyte differentiation into dendritic cells [39]. Interestingly, HLA-G exerts its immune tolerogenic function towards T cell alloproliferation following an independent pathway from IDO [40]. Nitric oxide-dependent nitration of both cellular and soluble HLA-G protein decreases total HLA-G cellular protein content and expression on the cell surface, while it increases HLA-G shedding into the culture medium. This effect was posttranscriptional and the result of metalloprotease activity [41–43]. Several evidences indicate that the soluble HLA-G1 (sHLA-G1) form is generated through the shedding of the membrane bound HLA-G1 by metalloproteinase (MP) [44–47]. In particular, matrix metalloproteinase-2 (MMP-2), a zinc-containing and calcium-requiring endopeptidase known for the ability to cleave several extracellular matrix constituents, as well as nonmatrix proteins, is responsible for HLA-G1 membrane-shedding via three possible highly specific cleavage sites [48].

The anti-inflammatory and immunosuppressive interleukin- (IL-) 10 has been correlated with concomitant HLA-G expression [33]. Transactivation of HLA-G transcription has also been demonstrated by leukemia inhibitory factor (LIF) [49] and methotrexate cell exposure [50]. Furthermore, interferon (IFN)-*α*, -**β**, and -*γ* enhance HLA-G cell-surface expression by tumors or monocytes [51, 52]. HLA-G expression could be acquired by trogocytosis, where a “donor” cell that expresses membrane HLA-G exchanges membrane parts containing HLA-G with a “recipient” cell that is not expressing HLA-G molecules. In this particular situation, “recipient” cells will acquire and make use of membrane HLA-G molecules from a “donor” HLA-G positive cell without the activation of HLA-G gene transduction into protein. Trogocytosis of HLA-G from antigen presenting cell (APC) by T cells in humans makes these T cells unresponsive [53]. It has been shown that NK cells can acquire HLA-G1 from tumor cells, which provokes an arrest of NK cells proliferation and cytotoxic activity, behaving like suppressor cells capable of inhibiting other NK cell functions [54].

## 3. HLA-G Transcription Products

**Figure 2:**
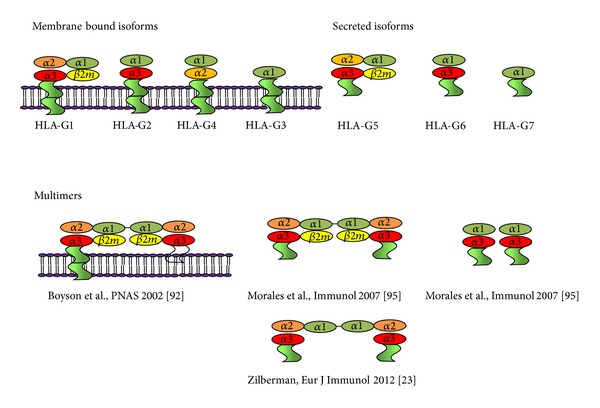
HLA-G isoforms and conformations. Membrane and soluble HLA-G isoforms are reported as monomeric and dimeric conformation Zilberman, Eur J Immunol 2012 [23].

To date, 50 alleles (IMGT HLA database, December 2013) and 16 proteins are known. Seven HLA-G isoforms exist due to mRNA alternative splicing and differential association with *β*2-microglobulin (*β*2-m). Four of them are found on the cell surface (HLA-G1, -G2, -G3, and -G4), while the other three are soluble forms released from the cell (HLA-G5, -G6, and -G7), due to the lack of the transmembrane and intracellular domains of membrane-bound HLA-G (Figure 2). The HLA-G 14 bp INS/DEL polymorphism is involved in the expression of both HLA-G1 and HLA-G5 isoforms, with decreased HLA-G1 and HLA-G5 concentrations in 14 bp INS samples in comparison with 14 bp DEL samples [32, 34].

The overall structure of HLA-G resembles other class I MHC molecules, in which a heavy chain comprised of three extracellular domains is noncovalently associated with *β*2-m (Figure 2). A nine-residue self-peptide is bound within a cleft formed by two alpha-helices and a beta-sheet floor. An extensive network of contacts is formed between the peptide and the binding cleft, leading to a constrained mode of binding reminiscent of that observed in HLA-E [65].

## 4. HLA-G Receptors

HLA-G exerts its immunomodulatory functions through the interaction with multiple receptors such as LILRB1 (ILT2/CD85j), LILRB2 (ILT4/CD85d), and KIR2DL4 (CD158d), which are differentially expressed by immune cells. The interaction of HLA-G molecules with inhibitory receptors induces apoptosis of activated CD8+ T cells [66], modulates the activity of NK cells [67] and dendritic cells (DC) [68], blocks allocytotoxic T lymphocyte response, induces expansion of T cell populations such as CD4^+^CD25^+^FoxP3^+^ regulatory T (Treg) cells [69] and CD3^+^CD4^low^Foxp3^−^ and CD3^+^CD8^low^Foxp3^−^ [70], and inhibits V*γ*9V*δ*2 T-cell proliferation and cytotoxicity without inducing apoptosis [71]. Moreover, HLA-G is expressed at high levels on DC-10 cells, human DCs with tolerogenic activity and an outstanding ability to produce IL-10 [72]. Interestingly, the expression of membrane-bound HLA-G1 and its receptors is upregulated by IL-10 on DC-10 and the expression of high levels of membrane-bound HLA-G1, ILT4, and IL-10 by DC-10 is critical to the generation of allergen-specific Tr1 cells by DC-10.

Whereas LILRB1 is expressed by NK cells, T cells, DCs, and decidual macrophages, LILRB2 expression is restricted to monocytes, macrophages, and DCs. These receptors can bind both classical and nonclassical HLA-I molecules [73, 74]. However, they present more affinity for HLA-G than for classical HLA-I molecules [75]. Also, HLA-G interaction with LILRB1 on NK cells and the resultant inhibitory function do not require tumor cell lipid raft integrity [76]. This differs from classical HLA-I, which are recruited in lipid rafts upon receptor engagement [77].

LILRB1 and LILRB2 possess 4 extracellular domains (D1–D4) and four and three immunoreceptor tyrosine-based inhibitory motifs (ITIMs), respectively, in their long cytoplasmic tails. These ITIM motifs confer them inhibitory characteristics, contrary to other LILR family receptors with activating properties that lack these ITIM motifs and possess an Arg residue in the transmembrane domain [74]. Interaction of LILRB1 and LILRB2 with their ligands causes phosphorylation of these ITIMS and recruitment of SHP phosphatases that initiate the inhibitory cascade. The D1 and D2 domains mediate the interaction of these receptors with HLA-I molecules and in the case of LILRB1 that occurs with the *α*3-domain and *β*2-m [74]. In fact, *β*2-m free HLA-G molecules are not recognized by LILRB1 [78]. However, in the case of LILRB2, it seems that interactions of these receptors with HLA-I molecules implicate the conservative residues of *α*3-domain but not of *β*2-m [73, 74]. HLA-G can form dimers that bind to LILR receptors with even a higher affinity than HLA-G monomers [79], being able to bind two receptors simultaneously [80].

Another HLA-G receptor is KIR2DL4 or CD158d, the only receptor of the killer cell immunoglobulin-like receptors (KIR) family that is expressed in all NK cell types [67]. KIR family includes receptors with activating properties and receptors with inhibitory properties. KIR2DL4 has unique structural properties among the rest of KIR receptors: it possesses a long cytoplasmic tail characteristic of inhibitory receptors, a charged amino acid in the transmembrane domain similarly to activating KIR receptors (reviewed [81]), and a mixed structure in the extracellular part with D0 and D2 domains. Contrary to other KIR receptors, KIR2DL4 expression is transitory on NK cell surface, with a main expression in endosomes, reached by an endocytic process. KIR2DL4 seems to participate to HLA-G endocytosis when it is transient expressed on NK cell surface, as both HLA-G and KIR2DL4 can be simultaneously colocalized in endosomes [82]. This could explain why soluble HLA-G or anti-KIR2DL4 antibodies, but not solid-phase bound antibodies, can induce cytokine secretion by NK resting cells. However, KIR2DL4 expression can be induced by IL-2 and its activation upon antibodies engagement provokes a weak cytotoxic activity with a strong IFN-*γ* production [83].

In vitro studies have shown that KIR2DL4 is able to interact with *β*2-m free HLA-G molecules, inducing IFN-*γ* production [84] and increasing NK cell cytotoxicity [19]. Contrary to LILR receptors, KIR does not bind HLA-I molecules through its *α*3 domain but through *α*1 and *α*2 domains which are much more polymorphic than *α*3 domain [85, 86]. This could account for the broader specificity of LILR receptors in comparison with KIR2DL4 that binds specifically HLA-G and no other HLA-I molecules. Also, structural studies suggest that KIR2DL4 cannot bind HLA-G dimers due to steric reasons [22].

The expression of LILRB1, LILRB2, and KIR2DL4 can be induced by HLA-G without any costimulatory requirement, which indicates that it can occur independently from any immune response [87].

Besides these receptors, HLA-G can also bind to CD8 without TCR interaction, provoking NK cells and activated CD8+ T cells apoptosis, and FasL upregulation and secretion [88]. Another putative HLA-G receptor is CD160. Interaction of HLA-G with CD160 expressed by endothelial cells induces the apoptosis of these cells [89] and inhibits cell proliferation, migration, and tubule formation [90], inhibiting the angiogenic process.

## 5. Posttranslational Modifications of HLA-G Molecule

Although most studies are related to *β*2-m bound HLA-G molecules that correspond to the originally described structure, several results have demonstrated the existence of modified variants of this structure. For example, expression of *β*2-m free HLA-G, which can be originated by dissociation of HLA-G complete isoforms [45], has been demonstrated in different tissues such as placenta [78] or pancreatic endocrine cells [91].

Besides Cys residues in *α*2 and *α*3 domains that allow intramolecular disulphide bonds, HLA-G molecule presents other important Cys residues. Cys42 in *α*1 domain and Cys147 in *α*2 domain can form intermolecular disulphide bonds giving rise to HLA-G dimers that can be observed by SDS/PAGE under nonreducing conditions [92]. These structures have been observed for all HLA-G isoforms except HLA-G3 [93]. It has been estimated that about 40% of HLA-G molecules at trophoblastic cells surface are in a dimeric form; meanwhile, only a small fraction of soluble HLA-G would be constituted by HLA-G dimers [94]. Even more, villous cytotrophoblast cells can produce dimers of *β*2-m free HLA-G5 molecules [95].

Immunoblot analysis with 4H84 antibody rends bands of diverse molecular weights (35–50 kDa) due to a glycosylation of HLA-G through an Asn residue (Asn86). This modification has been observed for both soluble and membrane bound HLA-G [96]. Another posttranslational modification observed in HLA-G is nitration in Tyr residues. Presence of 3-nitrotryrosine in HLA-G has been demonstrated in vivo in biological fluids both in monomeric and multimeric form [42] and in vitro after treatment with NO donors, which also increase HLA-G shedding by metalloproteases [43]. The detection of this modified HLA-G may characterize HLA-G synthesized at sites of inflammation where there is an important peroxide production.

Recently, HLA-G of molecular weights (70–76 kDa) higher than those expected were observed in biological fluids even when SDS/PAGE prior to western blot was performed under reducing conditions [64]. These molecules were associated with *β*2-m and could form dimers through disulphide bonds. The importance of these structures resides in the fact that they are not equivalently recognized by anti-HLA-G antibodies and can originate discrepancies in HLA-G quantification results. These molecules were later identified as ubiquitinated HLA-G molecules [97] with an intracellular origin demonstrated by their presence in exosomes, which are microvesicles of 50–100 nm originated from the endolysosomal pathway and secreted by many different cell types [98].

These particles carries mRNA, miRNA, and proteins, such as classical HLA-I molecules [98], and can exert distant immune functions [98]. Exosomes could act as a mechanism to spread HLA-G tolerogenic functions because HLA-G presence has been demonstrated in exosomes produced by melanoma cells [99] and by early and term placenta [100]. Furthermore, in serum from pregnant women HLA-G can be detected incorporated into exosomes [101].

## 6. Analytical Challenging in Soluble HLA-G Analysis

Searching in PubMed with the words HLA-G and ELISA there are 175 papers published until November, 2013, measuring soluble HLA-G in different biological fluids, including serum, plasma, and exudates. From these papers, it is clear that the measurement of soluble HLA-G is a potential biomarker for diagnostic and/or prognostic in some physiopathological situations, such as obstetric complications or cancer [102]. In addition detectable levels of soluble HLA-G in medium from embryo culture are associated with success in vitro fertilization. For this reason, the disposal of a good and widely accepted method to measure the soluble HLA-G levels is of crucial importance to achieve a good translation of results between different laboratories. Most are in-house ELISA assays (Table 1) using as capture antibody the mAb MEM-G/9, which has been raised against recombinant human HLA-G refolded with *β*2-m and peptide [56]. Other ELISAs are designed to measure exclusively HLA-G5 and/or -G6 using anti-pan HLA-I antibody W6/32 as detection antibody and the antibody 5A6G7 as capture antibody [103], that reacts with the intron 4, which is exclusive of these two isoforms [104]. As detection antibody most assays use an anti-**β**2-m antibody or W6/32. These assays perform very well in vitro using cell cultures, but the procedure for HLA-G measurement is far from being resolved in vivo, and it has been a source of conflicting results and interesting discussions [105–108]. More than 15 years have passed since first reports of a method for measuring HLA-G [55] and meanwhile some important efforts have been carried out by several authors to validate a method and a standard to measure soluble HLA-G [58]. However, four main problems remain to be solved: the identification of the main circulating HLA-G molecules in vivo, the obtaining of a purified standard widely available, the selection of the antibodies used in the procedure, and the sensitivity of the methodology.

Probably the most important issue is related to the types of HLA-G molecules present in biological fluids, as we do not know yet the predominant isoform and whether it circulates free or included in microvesicles, that is, exosomes [64], or if they are mainly free molecules or associated with **β**2-m, or even the influence of modifications such as dimerization [92], nitration [42], or ubiquitination [97]. The presence of these altered structures could be more relevant in cancer where there is a deeply altered microenvironment. Probably, the predominant structures in biological fluids are the dimeric or multimeric forms, considering that the extracellular redox status is more oxidized than the redox status and that there is a low proportion of free SH groups from the Cys in circulation [41]. It is not known if these proteins react equally with different antibodies employed to measure HLA-G in ELISA. Assuming the statement that only shed HLA-G1 and HLA-G5 are released to circulation, we and others have calculated the amount of sHLA-G1 by the difference between the concentrations of sHLA-G1/HLA-G5 (using MEM-G/9 as capture mAb) and HLA-G5 (using 5A6G7 as capture mAb) [109]. However, under the new vision of circulating HLA-G molecules we cannot be sure now that this always occurs in vivo. Unexpected results probably due to anomalous structures were already documented in the Wet-Workshop for Quantification of Soluble HLA-G held in 2004 [58]. In this workshop it was observed in some samples that there were HLA-G 5A6G7-immunoreactive molecules that were not recognized by MEM-G/9. These different structures were later elucidated to be new high molecular weight HLA-G complexes [64].

A second important problem is the lack of a widely available purified HLA-G molecule that could serve as a standard. The only commercial soluble HLA-G available kit nowadays for quantitative measurement (EXBIO Praha, Czech Republic) uses a sHLA-G standard calibrator in terms of arbitrary units/mL, but its equivalence to a protein concentration or biological activity is unknown. A high useful method to produce a protein is by plasmid transfection in bacteria, and both HLA-G1 and a fusion protein have been produced by this methodology [110]. As synthetized in a prokaryotic model, there are not the posttranslational modifications produced in eukaryotes, mainly glycosylation [96], and probably their conformation is not equivalent to the native protein. For example, the fusion proteins produce inhibition in NK cells only at levels much higher than the native protein. HLA-G5 molecules purified from detergent lysates of SF9 cells transfected with HLA-G5 and human *β*2-m have been used as a standard [58], while others have purified the protein from HLA-G transfected cell culture supernatants by affinity chromatography [111]. Also, other studies use dilutions of tested cell supernatants as standard, but the concentrations obtained cannot be extrapolated to other studies [109]. A standard widely available that could serve for data comparison between different laboratories could be of interest, so data could be transferred between papers. Until this standard becomes available, HLA-G level comparisons between different laboratories should be taken with caution. Same precautions should be taken when transferring the reference values that depend on not only both the standard and methodology used, but also on the population studied.

The third issue is related to the capability of the antibodies to recognize all forms of HLA-G. Most of the anti-HLA-G antibodies used in the ELISA recognize the native protein, are very specific, and do not react with other HLA-I molecules (Table 1) as it has been discussed elsewhere [112]. We do not know yet if the reaction is equimolar with all HLA-G molecules, and probably some proteins could be underrecognized. For example, it was recently shown by flow cytometry that MEM-G/9 can also react with HLA-G3, but the intensity of the signal is weaker than with HLA-G1 [113]. Some HLA-G complexes are underrecognized by MEM-G/9 and react better with the anti-HLA-G antibody G-233 [64]. In addition, although HLA-G polymorphism is quite low with only 16 proteins described to date, we do not know yet how they affect the binding to the antibodies. Of particular interest is that although the capture antibody in ELISA is HLA-G specific, only a few authors have used a specific antibody for HLA-G as detection antibody [63]. Instead, as we mentioned before, the detection antibody used in most of the ELISAs is an anti-**β**2-m antibody. The fact that HLA-G1 and -G5 from cell cultures are complexed with *β*2-m does not imply that the same occurs always in vivo in all clinical situations. Some of HLA-G released by the embryo is not bound to **β**2-m [95], so these molecules would not be detected in this type of assays. Although some authors have used the anti-HLA-G mAb 4H84 in ELISA [114], its use is not recommended as it can produce some nonspecific reactions with classical HLA-I molecules, under certain methodological conditions [113].

Finally, another problem to be solved is the sensitivity of the method. An important issue is that neither the functional sensitivity nor the analytical sensitivity is usually reported. Most methods are sandwich-ELISAs with colorimetric detection, whose reported detection limit is in the order of 1–10 ng/mL and soluble HLA-G levels are below this detection limit in many occasions. Thus, it is not known if there is no circulating HLA-G or if the procedure is not sensitive enough for quantification of low HLA-G levels. Some authors have improved the methodology, using fluorescence detection or with procedures based on microspheres technology. The detection limit decreased one order of magnitude compared to the colorimetric based ELISA methodology [59, 61]. This last methodology seems more appropriate for measuring HLA-G in media from embryo culture during in vitro fertilization [59].

## 7. Conclusions

HLA-G is a molecule that has been deeply studied during the last two decades where the almost exclusive expression in placenta has been well documented. When the HLA-G gene is expressed, it can produce seven isoforms that exert immune-suppressive functions by binding to its receptors. However, there are some important basic concepts in its biochemistry that remain not well explained yet. Among them, the knowledge of the regulation of the protein expression is a corner stone to understand how it can be expressed ectopically in different pathological situations. This could help to induce HLA-G in a tissue when a suppressive action is convenient (e.g., organ transplantation) or to suppress it when its expression is harmful (e.g., tumor). Also, along recent years multiple HLA-G protein modifications have been described, such as HLA-G dimers that bind LILRB receptors with an affinity even higher than monomers, or nitration. Moreover, high molecular weight molecules of HLA-G have been described as HLA-G complexed with ubiquitin. Furthermore, circulating HLA-G has also been observed as included in exosomes. The complete identification of these circulating HLA-G structures would improve not only the knowledge of this molecule but also the design of better methods for analysis. These are important questions that should be elucidated in order to understand the biology of HLA-G and to clarify some discrepant results.

## Figures and Tables

**Table 1:** Examples of methods developed for measuring soluble HLA-G.

Type	Standard	Capture antibody	Detection antibody	Detection	Detection limit	Reference
ELISA-sandwich	HLA-G1/LCL 722.221 transfected cells	W6.32 after depletion with TP25.99	anti-β2-m	Colorimetric	2.1 ng/mL	[55]

ELISA-sandwich	None	87G, BFL.1 or MEM-G/9	W6/32	Colorimetric	O.D.	[56]

ELISA-sandwich	HLA-G transfected CHO cells	G233	56B	Colorimetric	1 ng/mL	[57]

ELISA-sandwich	HLA-G5 protein derived from insect SF9 cells	MEM-G/9	anti-β2-m	Colorimetric	5 ng/mL	[58]

ELISA-sandwich	HLA-G5 protein derived from insect SF9 cells	5A6G7	W6/32	Colorimetric	5 ng/mL	[58]

Luminex	HLA-G5 and beta2 m transfected SF9 cells	MEM-G9	anti-β2-m	Fluorescence	0.3 ng/mL	[59]

ELISA-sandwich	HLA-G transfected LCL 721.221 cells	MEM-G/9	W6/32	Fluorescence	1 ng/mL	[60]

Bio-Plex	HLA-G5 transfected HeLa cells	MEM-G/9	W6/32	Fluorescence	0.3 ng/mL	[61]

ELISA-sandwich	HLA-G transfected LCL 721.221 cells	MEM-G/9	W6/32	Chemiluminescence	2 ng/mL	[62]

ELISA-sandwich	Purified HLA-G	HGY (noncommercial)	Polyclonal anti-HLA-G	Colorimetric	1 U/mL	[63]

ELISA-sandwich	HLA-G1 transfected LCL-721.221 cells	G233	anti-β2-m	Colorimetric	4 ng/mL	[64]

## References

[1] Hara N, Fujii T, Yamashita T, Kozuma S, Okai T, Taketani Y (1996). Altered expression of human leukocyte antigen G (HLA-G) on extravillous trophoblasts in preeclampsia: immunohistological demonstration with anti-HLA-G specific antibody “87G” and anti-cytokeratin antibody ‘CAM5.2’. *The American Journal of Reproductive Immunology*.

[2] Yie S-M, Li L-H, Li Y-M, Librach C (2004). HLA-G protein concentrations in maternal serum and placental tissue are decreased in preeclampsia. *The American Journal of Obstetrics and Gynecology*.

[3] Peng B, Zhang L, Xing A-Y, Hu M, Liu S-Y (2008). The expression of human leukocyte antigen G and E on human first trimester placenta and its relationship with recurrent spontaneous abortion. *Sichuan Da Xue Xue Bao Yi Xue Ban*.

[4] Le Discorde M, Moreau P, Sabatier P, Legeais J-M, Carosella ED (2003). Expression of HLA-G in human cornea, an immune-privileged tissue. *Human Immunology*.

[5] Mallet V, Blaschitz A, Crisa L (1999). HLA-G in the human thymus: a subpopulation of medullary epithelial but not CD83+ dendritic cells expresses HLA-G as a membrane-bound and soluble protein. *International Immunology*.

[6] Blaschitz A, Lenfant F, Mallet V (1997). Endothelial cells in chorionic fetal vessels of first trimester placenta express HLA-G. *European Journal of Immunology*.

[7] Rizzo R, Andersen AS, Lassen MR (2009). Soluble human leukocyte antigen-G isoforms in maternal plasma in early and late pregnancy. *The American Journal of Reproductive Immunology*.

[8] Rebmann V, Busemann A, Lindemann M, Grosse-Wilde H (2003). Detection of HLA-G5 secreting cells. *Human Immunology*.

[9] Derrien M, Pizzato N, Dolcini G (2004). Human immunodeficiency virus 1 downregulates cell surface expression of the non-classical major histocompatibility class I molecule HLA-G1. *Journal of General Virology*.

[10] Matte C, Lajoie J, Lacaille J, Zijenah LS, Ward BJ, Roger M (2004). Functionally active HLA-G polymorphisms are associated with the risk of heterosexual HIV-1 infection in African women. *AIDS*.

[11] Onno M, Le Friec G, Pangault C (2000). Modulation of HLA-G antigens expression in myelomonocytic cells. *Human Immunology*.

[12] Donaghy L, Gros F, Amiot L (2007). Elevated levels of soluble non-classical major histocompatibility class I molecule human leucocyte antigen (HLA)-G in the blood of HIV-infected patients with or without visceral leishmaniasis. *Clinical and Experimental Immunology*.

[13] Pistoia V, Morandi F, Wang X, Ferrone S (2007). Soluble HLA-G: are they clinically relevant?. *Seminars in Cancer Biology*.

[14] Crispim JCO, Duarte RA, Soares CP (2008). Human leukocyte antigen-G expression after kidney transplantation is associated with a reduced incidence of rejection. *Transplant Immunology*.

[15] Sebti Y, Le Maux A, Gros F (2007). Expression of functional soluble human leucocyte antigen-G molecules in lymphoproliferative disorders. *British Journal of Haematology*.

[16] Lila N, Carpentier A, Amrein C, Khalil-Daher I, Dausset J, Carosella ED (2000). Implication of HLA-G molecule in heart-graft acceptance. *The Lancet*.

[17] Lila N, Amrein C, Guillemain R (2002). Human leukocyte antigen-G expression after heart transplantation is associated with a reduced incidence of rejection. *Circulation*.

[18] Qiu J, Terasaki PI, Miller J, Mizutani K, Cai J, Carosella ED (2006). Soluble HLA-G expression and renal graft acceptance. *The American Journal of Transplantation*.

[19] Zhao L, Purandare B, Zhang J, Hantash BM (2013). Beta2-Microglobulin-free HLA-G activates natural killer cells by increasing cytotoxicity and proinflammatory cytokine production. *Human Immunology*.

[20] Rizzo R, Hviid TVF, Govoni M (2008). HLA-G genotype and HLA-G expression in systemic lupus erythematosus: HLA-G as a putative susceptibility gene in systemic lupus erythematosus. *Tissue Antigens*.

[21] Rizzo R, Farina I, Bortolotti D (2013). HLA-G may predict the disease course in patients with early rheumatoid arthritis. *Human Immunology*.

[22] Clements CS, Kjer-Nielsen L, McCluskey J, Rossjohn J (2007). Structural studies on HLA-G: implications for ligand and receptor binding. *Human Immunology: Trends in HLA-G Research*.

[23] Zilberman S, Schenowitz C, Agaugué S (2012). HLA-G1 and HLA-G5 active dimers are present in malignant cells and effusions: the influence of the tumor microenvironment. *European Journal of Immunology*.

[24] Solier C, Mallet V, Lenfant F, Bertrand A, Huchenq A, Le Bouteiller P (2001). HLA-G unique promoter region: functional implications. *Immunogenetics*.

[25] Gobin SJP, Keijsers V, Cheong C, van Zutphen M, van den Elsen PJ (1999). Transcriptional regulation of HLA-G. *Transplantation Proceedings*.

[26] Gobin SJP, van Zutphen M, Woltman AM, van den Elsen PJ (1999). Transactivation of classical and nonclassical HLA class I genes through the IFN-stimulated response element. *Journal of Immunology*.

[27] Lefebvre S, Berrih-Aknin S, Adrian F (2001). A specific interferon (IFN)-stimulated response element of the distal HLA-G promoter binds IFN-regulatory factor 1 and mediates enhancement of this nonclassical class I gene by IFN-*β*. *Journal of Biological Chemistry*.

[28] Ibrahim EC, Morange M, Dausset J, Carosella ED, Paul P (2000). Heat shock and arsenite induce expression of the nonclassical class I histocompatibility HLA-G gene in tumor cell lines. *Cell Stress and Chaperones*.

[29] Yie S-M, Xiao R, Librach CL (2006). Progesterone regulates HLA-G gene expression through a novel progesterone response element. *Human Reproduction*.

[30] Castelli EC, Mendes-Junior CT, Veiga-Castelli LC, Roger M, Moreau P, Donadi EA (2011). A comprehensive study of polymorphic sites along the HLA-G gene: implication for gene regulation and evolution. *Molecular Biology and Evolution*.

[31] Ober C, Billstrand C, Kuldanek S, Tan Z (2006). The miscarriage-associated HLA-G -725G allele influences transcription rates in JEG-3 cells. *Human Reproduction*.

[32] Hviid TVF, Hylenius S, Rørbye C, Nielsen LG (2003). HLA-G allelic variants are associated with differences in the HLA-G mRNA isoform profile and HLA-G mRNA levels. *Immunogenetics*.

[33] Hviid TVF, Rizzo R, Christiansen OB, Melchiorri L, Lindhard A, Baricordi OR (2004). HLA-G and IL-10 in serum in relation to HLA-G genotype and polymorphisms. *Immunogenetics*.

[34] Svendsen SG, Hantash BM, Zhao L (2013). The expression and functional activity of membrane-bound human leukocyte antigen-G1 are influenced by the 3′-untranslated region. *Human Immunology*.

[35] Yie S-M, Li L-H, Xiao R, Librach CL (2008). A single base-pair mutation in the 3′-untranslated region of HLA-G mRNA is associated with pre-eclampsia. *Molecular Human Reproduction*.

[36] Tan Z, Randall G, Fan J (2007). Allele-specific targeting of microRNAs to HLA-G and risk of asthma. *The American Journal of Human Genetics*.

[37] Manaster I, Goldman-Wohl D, Greenfield C (2012). MiRNA-mediated control of HLA-G expression and function. *PLoS ONE*.

[38] Mouillot G, Marcou C, Zidi I (2007). Hypoxia modulates HLA-G gene expression in tumor cells. *Human Immunology*.

[39] López AS, Alegre E, LeMaoult J, Carosella E, González Á (2006). Regulatory role of tryptophan degradation pathway in HLA-G expression by human monocyte-derived dendritic cells. *Molecular Immunology*.

[40] Le Rond S, Gonzalez A, Gonzalez ASL, Carosella ED, Rouas-Freiss N (2005). Indoleamine 2,3 dioxygenase and human leucocyte antigen-G inhibit the T-cell alloproliferative response through two independent pathways. *Immunology*.

[41] Díaz-Lagares A, Alegre E, Arroyo A, Corrales FJ, González Á (2009). Tyrosine nitration in the human leucocyte antigen-G-binding domain of the Ig-like transcript 2 protein. *The FEBS Journal*.

[42] Díaz-Lagares Á, Alegre E, Gonzalez Á (2009). Detection of 3-nitrotyrosine-modified human leukocyte antigen-G in biological fluids. *Human Immunology*.

[43] Díaz-Lagares A, Alegre E, LeMaoult J, Carosella ED, González Á (2009). Nitric oxide produces HLA-G nitration and induces metalloprotease-dependent shedding creating a tolerogenic milieu. *Immunology*.

[44] Park GM, Lee S, Park B (2004). Soluble HLA-G generated by proteolytic shedding inhibits NK-mediated cell lysis. *Biochemical and Biophysical Research Communications*.

[45] Dong Y, Lieskovska J, Kedrin D, Porcelli S, Mandelboim O, Bushkin Y (2003). Soluble nonclassical HLA generated by the metalloproteinase pathway. *Human Immunology*.

[46] Demaria S, Schwab R, Gottesman SRS, Bushkin Y (1994). Soluble *β*2-microglobulin-free class I heavy chains are released from the surface of activated and leukemia cells by a metalloprotease. *Journal of Biological Chemistry*.

[47] Zidi I, Guillard C, Marcou C (2006). Increase in HLA-G1 proteolytic shedding by tumor cells: a regulatory pathway controlled by NF-*κ*B inducers. *Cellular and Molecular Life Sciences*.

[48] Rizzo R, Trentini A, Bortolotti D (2013). Matrix metalloproteinase-2 (MMP-2) generates soluble HLA-G1 by cell surface proteolytic shedding. *Molecular and Cellular Biochemistry*.

[49] Bamberger A-M, Henatschke S, Schulte HM, Löning T, Bamberge CM (2000). Leukemia inhibitory factor (LIF) stimulates the human HLA-G promoter in JEG3 choriocarcinoma cells. *Journal of Clinical Endocrinology and Metabolism*.

[50] Rizzo R, Rubini M, Govoni M (2006). HLA-G 14-bp polymorphism regulates the methotrexate response in rheumatoid arthritis. *Pharmacogenetics and Genomics*.

[51] Ugurel S, Rebmann V, Ferrone S, Tilgen W, Grosse-Wilde H, Reinhold U (2001). Soluble human leukocyte antigen-G serum level is elevated in melanoma patients and is further increased by interferon-alpha immunotherapy. *Cancer*.

[52] Lefebvre S, Moreau P, Guiard V (1999). Molecular mechanisms controlling constitutive and IFN-*γ*-inducible HLA-G expression in various cell types. *Journal of Reproductive Immunology*.

[53] LeMaoult J, Caumartin J, Daouya M (2007). Immune regulation by pretenders: cell-to-cell transfers of HLA-G make effector T cells act as regulatory cells. *Blood*.

[54] Caumartin J, Favier B, Daouya M (2007). Trogocytosis-based generation of suppressive NK cells. *The EMBO Journal*.

[55] Rebmann V, Pfeiffer K, Pssler M (1999). Detection of soluble HLA-G molecules in plasma and amniotic fluid. *Tissue Antigens*.

[56] Fournel S, Huc X, Aguerre-Girr M (2000). Comparative reactivity of different HLA-G monoclonal antibodies to soluble HLA-G molecules. *Tissue Antigens*.

[57] van Lierop MJC, Wijnands F, Loke YW (2002). Detection of HLA-G by a specific sandwich ELISA using monoclonal antibodies G233 and 56B. *Molecular Human Reproduction*.

[58] Rebmann V, LeMaoult J, Rouas-Freiss N, Carosella ED, Grosse-Wilde H (2007). Quantification and identification of soluble HLA-G isoforms. *Tissue Antigens*.

[59] Rebmann V, Switala M, Eue I, Schwahn E, Merzenich M, Grosse-Wilde H (2007). Rapid evaluation of soluble HLA-G levels in supernatants of in vitro fertilized embryos. *Human Immunology*.

[60] Sageshima N, Shobu T, Awai K (2007). Soluble HLA-G is absent from human embryo cultures: a reassessment of sHLA-G detection methods. *Journal of Reproductive Immunology*.

[61] Rizzo R, Dal Canto MB, Stignani M (2009). Production of sHLA-G molecules by in vitro matured cumulus-oocyte complex. *International Journal of Molecular Medicine*.

[62] Tabiasco J, d’Hauterive SP, Thonon F (2009). Soluble HLA-G in IVF/ICSI embryo culture supernatants does not always predict implantation success: a multicentre study. *Reproductive BioMedicine*.

[63] Cao M, Yie S-M, Liu J, Ye SR, Xia D, Gao E (2011). Plasma soluble HLA-G is a potential biomarker for diagnosis of colorectal, gastric, esophageal and lung cancer. *Tissue Antigens*.

[64] Gonzalez Á, Alegre E, Arroyo A, LeMaoult J, Echeveste JI (2011). Identification of circulating nonclassic human leukocyte antigen G (HLA-G): like molecules in exudates. *Clinical Chemistry*.

[65] Clements CS, Kjer-Nielsen L, Kostenko L (2005). Crystal structure of HLA-G: a nonclassical MHC class I molecule expressed at the fetal-maternal interface. *Proceedings of the National Academy of Sciences of the United States of America*.

[66] Kapasi K, Albert SE, Yie S-M, Zavazava N, Librach CL (2000). HLA-G has a concentration-dependent effect on the generation of an allo-CTL response. *Immunology*.

[67] Rajagopalan S, Long EO (1999). A human histocompatibility leukocyte antigen (HLA)-G-specific receptor expressed on all natural killer cells. *Journal of Experimental Medicine*.

[68] Liang S, Ristich V, Arase H, Dausset J, Carosella ED, Horuzsko A (2008). Modulation of dendritic cell differentiation by HLA-G and ILT4 requires the IL-6: STAT3 signaling pathway. *Proceedings of the National Academy of Sciences of the United States of America*.

[69] Selmani Z, Naji A, Zidi I (2008). Human leukocyte antigen-G5 secretion by human mesenchymal stem cells is required to suppress T lymphocyte and natural killer function and to induce CD4+ CD25highFOXP3+ regulatory T cells. *Stem Cells*.

[70] Naji A, Le Rond S, Durrbach A (2007). CD3+CD4low and CD3+CD8low are induced by HLA-G: novel human peripheral blood suppressor T-cell subsets involved in transplant acceptance. *Blood*.

[71] Lesport E, Baudhuin J, Sousa S (2011). Inhibition of human gamma delta T-cell antitumoral activity through HLA-G: implications for immunotherapy of cancer. *Cellular and Molecular Life Sciences*.

[72] Gregori S, Tomasoni D, Pacciani V (2010). Differentiation of type 1 T regulatory cells (Tr1) by tolerogenic DC-10 requires the IL-10-dependent ILT4/HLA-G pathway. *Blood*.

[73] Shiroishi M, Kuroki K, Rasubala L (2006). Structural basis for recognition of the nonclassical MHC molecule HLA-G by the leukocyte Ig-like receptor B2 (LILRB2/LIR2/ILT4/CD85d). *Proceedings of the National Academy of Sciences of the United States of America*.

[74] Brown D, Trowsdale J, Allen R (2004). The LILR family: modulators of innate and adaptive immune pathways in health and disease. *Tissue Antigens*.

[75] Shiroishi M, Tsumoto K, Amano K (2003). Human inhibitory receptors Ig-like transcript 2 (ILT2) and ILT4 compete with CD8 for MHC class I binding and bind preferentially to HLA-G. *Proceedings of the National Academy of Sciences of the United States of America*.

[76] Baudhuin J, Lesport E, Sousa S (2012). HLA-G inhibition of NK-cell cytolytic function is uncoupled from tumor cell lipid raft reorganization. *European Journal of Immunology*.

[77] Yang J, Zhang X, Wang J (2007). Anti-*β*2-microglobulin monoclonal antibodies induce apoptosis in myeloma cells by recruiting MHC class I to and excluding growth and survival cytokine receptors from lipid rafts. *Blood*.

[78] Gonen-Gross T, Achdout H, Arnon TI (2005). The CD85J/leukocyte inhibitory receptor-1 distinguishes between conformed and *β*2-microglobulin-free HLA-G molecules. *Journal of Immunology*.

[79] Gonen-Gross T, Achdout H, Gazit R (2003). Complexes of HLA-G protein on the cell surface are important for leukocyte Ig-like receptor-1 function. *Journal of Immunology*.

[80] Shiroishi M, Kuroki K, Ose T (2006). Efficient leukocyte Ig-like receptor signaling and crystal structure of disulfide-linked HLA-G dimer. *Journal of Biological Chemistry*.

[81] Faure M, Long EO (2002). KIR2DL4 (CD158d), an NK cell-activating receptor with inhibitory potential. *Journal of Immunology*.

[82] Rajagopalan S, Bryceson YT, Kuppusamy SP (2006). Activation of NK cells by an endocytosed receptor for soluble HLA-G. *PLoS Biology*.

[83] Kikuchi-Maki A, Yusa S-I, Catina TL, Campbell KS (2003). KIR2DL4 is an IL-2-regulated NK cell receptor that exhibits limited expression in humans but triggers strong IFN-*γ* production. *Journal of Immunology*.

[84] Rajagopalan S, Fu J, Long EO (2001). Cutting edge: induction of IFN-*γ* production but not cytotoxicity by the killer cell Ig-like receptor KIR2DL4 (CD158d) in resting NK cells. *Journal of Immunology*.

[85] Riteau B, Rouas-Freiss N, Menier C, Paul P, Dausset J, Carosella ED (2001). HLA-G2, -G3, and -G4 isoforms expressed as nonmature cell surface glycoproteins inhibit NK and antigen-specific CTL cytolysis. *Journal of Immunology*.

[86] Gonen-Gross T, Goldman-Wohl D, Huppertz B (2010). Inhibitory NK receptor recognition of HLA-G: regulation by contact residues and by cell specific expression at the fetal-maternal interface. *PLoS ONE*.

[87] LeMaoult J, Zafaranloo K, Le Banff C, Carosella ED (2005). HLA-G up-regulates ILT2, ILT3, ILT4, and KIR2DL4 in antigen presenting cells, NK cells, and T cells. *The FASEB Journal*.

[88] Contini P, Ghio M, Poggi A (2003). Soluble HLA-A,-B,-C and -G molecules induce apoptosis in T and NK CD8+ cells and inhibit cytotoxic T cell activity through CD8 ligation. *European Journal of Immunology*.

[89] Fons P, Chabot S, Cartwright JE (2006). Soluble HLA-G1 inhibits angiogenesis through an apoptotic pathway and by direct binding to CD160 receptor expressed by endothelial cells. *Blood*.

[90] Le Bouteiller P, Fons P, Herault J-P (2007). Soluble HLA-G and control of angiogenesis. *Journal of Reproductive Immunology*.

[91] Cirulli V, Zalatan J, McMaster M (2006). The class I HLA repertoire of pancreatic islets comprises the nonclassical class Ib antigen HLA-G. *Diabetes*.

[92] Boyson JE, Erskine R, Whitman MC (2002). Disulfide bond-mediated dimerization of HLA-G on the cell surface. *Proceedings of the National Academy of Sciences of the United States of America*.

[93] HoWangYin KY, Loustau M, Wu J (2012). Multimeric structures of HLA-G isoforms function through differential binding to LILRB receptors. *Cellular and Molecular Life Sciences*.

[94] Apps R, Gardner L, Sharkey AM, Holmes N, Moffett A (2007). A homodimeric complex of HLA-G on normal trophoblast cells modulates antigen-presenting cells via LILRB1. *European Journal of Immunology*.

[95] Morales PJ, Pace JL, Platt JS, Langat DK, Hunt JS (2007). Synthesis of *β*2-microglobulin-free, disulphide-linked HLA-G5 homodimers in human placental villous cytotrophoblast cells. *Immunology*.

[96] McMaster M, Zhou Y, Shorter S (1998). HLA-G isoforms produced by placental cytotrophoblasts and found in amniotic fluid are due to unusual glycosylation. *Journal of Immunology*.

[97] Alegre E, Rebmann V, LeMaoult J (2013). In vivo identification of an HLA-G complex as ubiquitinated protein circulating in exosomes. *European Journal of Immunology*.

[98] Andre F, Schartz NEC, Movassagh M (2002). Malignant effusions and immunogenic tumour-derived exosomes. *The Lancet*.

[99] Riteau B, Faure F, Menier C (2003). Exosomes bearing HLA-G are released by melanoma cells. *Human Immunology*.

[100] Kshirsagar SK, Alam SM, Jasti S (2012). Immunomodulatory molecules are released from the first trimester and term placenta via exosomes. *Placenta*.

[101] Taylor DD, Akyol S, Gercel-Taylor C (2006). Pregnancy-associated exosomes and their modulation of T cell signaling. *Journal of Immunology*.

[102] Gonzalez A, Rebmann V, LeMaoult J, Horn PA, Carosella ED, Alegre E (2012). The immunosuppressive molecule HLA-G and its clinical implications. *Critical Reviews in Clinical Laboratory Sciences*.

[103] Rebmann V, LeMaoult J, Rouas-Freiss N, Carosella ED, Grosse-Wilde H (2005). Report of the wet workshop for quantification of soluble HLA-G in Essen, 2004. *Human Immunology*.

[104] Le Rond S, Le Maoult J, Créput C (2004). Alloreactive CD4+ and CD8+ T cells express the immunotolerant HLA-G molecule in mixed lymphocyte reactions: in vivo implications in transplanted patients. *European Journal of Immunology*.

[105] Blaschitz A, Juch H, Volz A, Hutter H, Dohr G (2005). Soluble HLA-G, the discussion is going on!. *Molecular Human Reproduction*.

[106] Sargent IL (2005). Does “soluble” HLA-G really exist? Another twist to the tale. *Molecular Human Reproduction*.

[107] LeMaoult J, Rouas-Freiss N, Carosella ED (2005). HLA-G5 expression by trophoblast cells: the facts. *Molecular Human Reproduction*.

[108] Hunt JS, Geraghty DE (2005). Soluble HLA-G isoforms: technical deficiencies lead to misinterpretations. *Molecular Human Reproduction*.

[109] Alegre E, Díaz-Lagares A, LeMaoult J, López-Moratalla N, Carosella ED, González A (2007). Maternal antigen presenting cells are a source of plasmatic HLA-G during pregnancy: longitudinal study during pregnancy. *Human Immunology*.

[110] Marchal-Bras-Goncalves R, Rouas-Freiss N, Connan F (2001). A soluble HLA-G protein that inhibits natural killer cell-mediated cytotoxicity. *Transplantation Proceedings*.

[111] Le Friec G, Laupèze B, Fardel O (2003). Soluble HLA-G inhibits human dendritic cell-triggered allogeneic T-cell proliferation without altering dendritic differentiation and maturation processes. *Human Immunology*.

[112] Apps R, Gardner L, Moffett A (2008). A critical look at HLA-G. *Trends in Immunology*.

[113] Zhao L, Teklemariam T, Hantash BM (2012). Reassessment of HLA-G isoform specificity of MEM-G/9 and 4H84 monoclonal antibodies. *Tissue Antigens*.

[114] Juch H, Blaschitz A, Daxböck C, Rueckert C, Kofler K, Dohr G (2005). A novel sandwich ELISA for *α*1 domain based detection of soluble HLA-G heavy chains. *Journal of Immunological Methods*.

